# Whole genome resequencing of black Angus and Holstein cattle for SNP and CNV discovery

**DOI:** 10.1186/1471-2164-12-559

**Published:** 2011-11-15

**Authors:** Paul Stothard, Jung-Woo Choi, Urmila Basu, Jennifer M Sumner-Thomson, Yan Meng, Xiaoping Liao, Stephen S Moore

**Affiliations:** 1 Department of Agricultural, Food and Nutritional Science, University of Alberta, Edmonton, AB T6G 2P5, Canada

## Abstract

**Background:**

One of the goals of livestock genomics research is to identify the genetic differences responsible for variation in phenotypic traits, particularly those of economic importance. Characterizing the genetic variation in livestock species is an important step towards linking genes or genomic regions with phenotypes. The completion of the bovine genome sequence and recent advances in DNA sequencing technology allow for in-depth characterization of the genetic variations present in cattle. Here we describe the whole-genome resequencing of two *Bos taurus *bulls from distinct breeds for the purpose of identifying and annotating novel forms of genetic variation in cattle.

**Results:**

The genomes of a Black Angus bull and a Holstein bull were sequenced to 22-fold and 19-fold coverage, respectively, using the ABI SOLiD system. Comparisons of the sequences with the Btau4.0 reference assembly yielded 7 million single nucleotide polymorphisms (SNPs), 24% of which were identified in both animals. Of the total SNPs found in Holstein, Black Angus, and in both animals, 81%, 81%, and 75% respectively are novel. In-depth annotations of the data identified more than 16 thousand distinct non-synonymous SNPs (85% novel) between the two datasets. Alignments between the SNP-altered proteins and orthologues from numerous species indicate that many of the SNPs alter well-conserved amino acids. Several SNPs predicted to create or remove stop codons were also found. A comparison between the sequencing SNPs and genotyping results from the BovineHD high-density genotyping chip indicates a detection rate of 91% for homozygous SNPs and 81% for heterozygous SNPs. The false positive rate is estimated to be about 2% for both the Black Angus and Holstein SNP sets, based on follow-up genotyping of 422 and 427 SNPs, respectively. Comparisons of read depth between the two bulls along the reference assembly identified 790 putative copy-number variations (CNVs). Ten randomly selected CNVs, five genic and five non-genic, were successfully validated using quantitative real-time PCR. The CNVs are enriched for immune system genes and include genes that may contribute to lactation capacity. The majority of the CNVs (69%) were detected as regions with higher abundance in the Holstein bull.

**Conclusions:**

Substantial genetic differences exist between the Black Angus and Holstein animals sequenced in this work and the Hereford reference sequence, and some of this variation is predicted to affect evolutionarily conserved amino acids or gene copy number. The deeply annotated SNPs and CNVs identified in this resequencing study can serve as useful genetic tools, and as candidates in searches for phenotype-altering DNA differences.

## Background

Cattle are an important source of meat, milk, and other goods in many parts of the world. Selective breeding has been used in conjunction with other approaches to increase the productivity of cattle and has contributed to dramatic changes in traits of interest. In dairy cattle, increases of 3,500 kg of milk, 130 kg of fat, and 100 kg of protein per cow per lactation have resulted from improvements in genetics, nutrition, and management during the past 20 years [1]. More than half of the increase in milk production in US Holstein cows achieved in the past 40 years is due to improved genetics [2]. Similarly, beef cattle have produced more meat of better quality than their recent ancestors due to selective breeding [2]. Considerable effort is also now focused on reducing the cost of raising animals by improving the efficiency of feed utilization [3]. Substantial gains in traits of interest have been made through selection of individuals for breeding based on their phenotypes, or those of their close relatives [4]. More recently, genomics technologies like SNP genotyping have been used to select animals on the basis of their genetic makeup [4]. Both of these methods can be applied without knowledge of the mechanisms that link the DNA variations to the traits. However, in addition to providing biological insights, identification of the specific DNA differences associated with these traits can be used to develop more accurate tools for genomic selection as well as non-breeding approaches for modifying traits.

A large catalogue of genetic variation, especially SNPs, exists for cattle in publicly accessible databases, thanks largely to the bovine HapMap project [5], the bovine genome project [6], and large-scale SNP discovery studies [7]. Nonetheless there is much genetic variation that remains to be discovered. Indeed, recent whole genome resequencing has revealed many novel SNPs [8], and a recent comparative genomic hybridization study has identified numerous candidate CNVs [9]. Continued characterization of genetic variation, particularly in breeds that have not been thoroughly scrutinized, will be an important step towards deciphering the molecular mechanisms underlying trait variation.

In this work we describe the whole-genome resequencing of two individuals from distinct cattle breeds for the purpose of identifying DNA differences. One of the sequenced animals, Goldwyn, is a bull from the Holstein breed. Holstein cattle originated from the Friesian breed in Europe and were likely first imported to North America in 1795 [10]. They are known for their black-and-white markings and high milk production, and are the main source of dairy products in North America. Goldwyn was produced by the Semex Alliance (Guelph, ON, Canada) in 2000, and became one of the top dairy sires in the world by virtue of his daughters' impressive characteristics. His semen, which currently sells for about $300 per straw, has been used to sire over 20,000 cows. The second animal is a Black Angus bull. The Black Angus breed originated in Scotland and was imported to North America in the late 1800s, where it is now the most popular beef breed. Thus the two animals characterized in this study are from distinct populations shaped by selection for distinct traits. In the case of the Holstein breed, selection has been especially intense as has been the rate of performance gains. We expect that these separate selection regimes have resulted in some different variants being favoured or fixed in each breed.

In our analyses of the Holstein and Black Angus sequences we used assembly version Btau4.0 [6] as a reference sequence. The Btau4.0 assembly was built using sequence from a Hereford cow and her sire [11]. The Hereford breed originated in Great Britain in the 1700s and currently is a popular breed for beef production in many parts of the world. A detailed SNP-based comparison of the Holstein, Black Angus, and Hereford breeds shows that each is genetically distinct [5]. SNPs were identified in this work as differences between the newly obtained genome sequences and the reference Hereford sequence, whereas potential CNVs were detected as regions of unequal read depth between the two resequenced animals. Detailed annotation of the results and downstream validation suggest the presence of many novel genetic variants, with several of the variants affecting evolutionarily conserved protein regions. The CNVs described in this work are enriched for immune system genes and genes that may contribute to lactation capacity. Most of the CNVs were detected as regions with higher abundance in the Holstein bull. The source and significance of this excess of CNV gains is not clear.

## Results

### Genome sequencing, SNP detection and SNP validation

Genomic DNA from a Black Angus bull and a Holstein bull were sequenced using the SOLiD system and a combination of fragment and mate pair libraries (Table 1). The resulting reads were mapped to a reference bovine genome assembly (Btau4.0) and yielded approximately 22-fold and 19-fold coverage for the two animals, respectively (Table 2). Putative SNPs were detected by comparing the aligned reads to the reference assembly. More than 3.7 million and 3.2 million SNPs were identified for the Holstein and Black Angus genomes, respectively.

**Table 1:** Sequencing libraries and sequencing runs

Library	Run name	Read length	F3 reads	R3 reads
Black Angus FR 50	solid_BA_FR1	50 nt	172,104,943	0

Black Angus FR 50	solid_BA_FR2	50 nt	225,062,253	0

Black Angus MP 25	solid_BA_MP1	25 nt	73,568,858	73,917,320

Black Angus MP 25	solid_BA_MP2	25 nt	76,123,158	76,573,521

Black Angus MP 25	solid_BA_MP3	25 nt	69,657,261	69,108,403

Black Angus MP 25	solid_BA_MP4	25 nt	356,566,421	356,848,504

Black Angus MP 50	solid_BA_MP5	50 nt	220,164,958	220,910,123

Black Angus MP 50	solid_BA_MP6	50 nt	78,350,806	80,494,794

Black Angus MP 50	solid_BA_MP7	50 nt	343,878,503	346,998,757

Holstein MP 50	solid_HOL_MP1	50 nt	140,503,099	142,438,241

Holstein MP 50	solid_HOL_MP2	50 nt	190,712,998	191,191,811

Holstein FR 50	solid_HOL_FR1	50 nt	172,127,298	0

Holstein FR 50	solid_HOL_FR2	50 nt	321,848,214	0

Holstein MP 25	solid_HOL_MP3	25 nt	160,118,556	159,430,437

Holstein MP 25	solid_HOL_MP4	25 nt	281,714,461	281,402,729

Three libraries were constructed for each animal and sequenced using two or more instrument runs. The numbers of reads obtained for fragment libraries is given in the F3 reads column. Mate-paired libraries yielded two read types (F3 reads and R3 reads).

**Table 2:** Coverage of the Holstein and Black Angus genomes

Genome	Total reads	Megabases of coverage	Fold coverage
Holstein	2,041,487,844	49,069.31	18.63

Black Angus	2,840,328,583	57,730.10	21.91

Megabases of coverage was calculated based on the numbers and lengths of reads that were successfully mapped to the reference. Fold coverage was calculated by dividing the megabases of coverage by the combined length of the reference chromosomes used for mapping (2,634,413,324 bp).

To estimate the rate at which SNPs were missed by sequencing (false negatives), the SNP list for the Holstein animal was compared to the genotypes obtained using an array-based genotyping assay. Of 226,854 homozygous array calls that were different from the reference (and thus would be detected by our SNP calling approach), 206,480 (91%) were identified as SNPs by sequencing, and 203,812 of the 226,854 (90%) SNPs showed concordant genotypes (Table 3). Based on these results we calculated the false negative rate for homozygous SNP detection as (1-203,812/226,854)*100 = 10%. Of the 189,784 heterozygous array calls, 152,910 (81%) were called as sequencing SNPs, and 149,550 (98%) of the 152,910 SNPs had concordant genotypes. From these results, we calculated the false negative rate for heterozygous SNP detection as (1-149,550/189,784)*100 = 21%. Examination of the discordant heterozygous calls reveals that the vast majority (98%) represent cases where the sequencing indicated the presence of just one of the alleles assayed on the array.

**Table 3:** Comparison of BovineHD array genotypes to sequencing SNPs

Detectable genotype	BovineHD	Sequencing calls	Concordant
Homozygous variant	226,854	206,480 (91%)	203,812 (90%)

Heterozygous	189,784	152,910 (81%)	149,550 (79%)

The sequenced Holstein animal was genotyped so that the ability of the sequencing to identify detectable SNPs (homozygous variant and heterozygote) could be quantified (false negative rate). The "Sequencing Calls" column gives the number of detectable array SNPs that were identified as SNPs through sequencing, regardless of whether the sequencing genotype matched the array genotype. The "Concordant" column gives the number of sequencing calls with a sequencing genotype that matched the array genotype.

To estimate the rate at which SNPs were called when no SNP was actually present, a custom genotyping assay was designed and applied. A group of 1083 animals was genotyped using 427 and 422 SNPs selected from the Holstein and Black Angus SNP lists, respectively. Of the 427 Holstein SNPs that were genotyped, 420 (98%) were found to be true SNPs (Table 4). Of the 422 Black Angus SNPs that were genotyped, 415 (98%) were demonstrated to be true SNPs (Table 4).

**Table 4:** Comparison of genotypes from a custom array to sequencing SNPs

Source of SNPs	SNPs tested	SNPs validated
Holstein	427	420 (98%)

Black Angus	422	415 (98%)

To estimate the false positive rate of SNP discovery, a subset of the SNPs discovered by sequencing was genotyped in 1083 animals.

### SNP annotation

The results of SNP annotation using NGS-SNP [12] suggest that the Holstein and Black Angus SNPs belong to a diverse range of functional classes. Most of the SNPs are located between genes or within introns (Table 5). A comparison of the SNPs identified in this work with those described in dbSNP build 130 [13] indicates that 81% of the SNPs detected in the Holstein animal, and 81% of the SNPs detected in the Black Angus animal, are novel. Subsets of the annotated SNPs are available in Additional file 1 and Additional file 2.

**Table 5:** SNP functional class membership

Functional class	Holstein	Black Angus	Intersection	Union
Intergenic	2,488,430(66.3)	2,131,566(65.7)	1,124,055(65.6)	3,495,941(66.1)

Intronic	1,003,805(26.7)	881,566(27.2)	469,082(27.4)	1,416,289(26.8)

Upstream	116,529(3.1)	103,589(3.2)	53,027(3.1)	167,091(3.2)

Downstream	104,762(2.8)	90,788(2.8)	48,128(2.8)	147,422(2.8)

Synonymous coding	16,161(0.4)	15,102(0.5)	8,051(0.5)	23,212(0.4)

Nonsynonymous coding	11,598(0.3)	10,723(0.3)	5,490(0.3)	16,831(0.3)

3' UTR	8,732(0.2)	7,753(0.2)	4,200(0.2)	12,285(0.2)

Splice site^a^	2,921(0.1)	2,679(0.1)	1,421(0.1)	4,179(0.1)

5' UTR	1,591(0.0)	1,382(0.0)	680(0.0)	2,293(0.0)

Within non coding gene	791(0.0)	763(0.0)	344(0.0)	1210(0.0)

Essential splice site^b^	197(0.0)	166(0.0)	94(0.0)	269(0.0)

Stop gained	126(0.0)	124(0.0)	46(0.0)	204(0.0)

Stop lost	13(0.0)	8(0.0)	5(0.0)	16(0.0)

Within mature miRNA	7(0.0)	2(0.0)	1(0.0)	8(0.0)

Total	3,755,663(100)	3,246,211(100)	1,714,624(100)	5,287,250(100)

^a^SNP is located 1-3 bases into an exon or 3-8 bases into an intron.

^b^SNP is located in the first two or the last two bases of an intron.

The predicted functional consequences of SNPs identified by sequencing of the Holstein and Black Angus genomes. Values in parentheses are the percentage of SNPs that are in the functional class, out of the total SNPs in the column.

**Figure 1:**
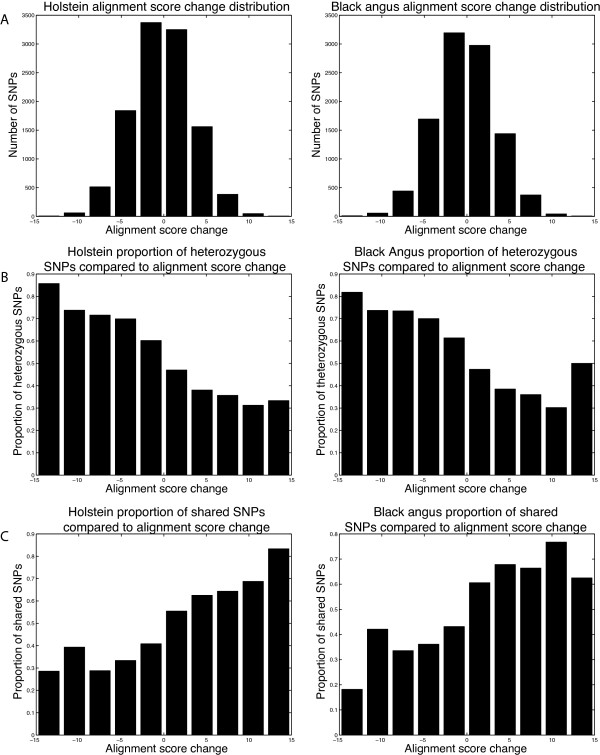
**Characteristics of nonsynonymous SNPs**. (A) Distribution of "alignment score change" for Holstein and Black Angus nonsynonymous SNPs generated using a bin size of 3. Negative scores indicate the presence of a non-reference-sequence allele that makes the protein less similar to its orthologues. Positive scores indicate the presence of a non-reference-sequence allele that makes the protein more similar to its orthologues. (B) Proportion of heterozygous SNPs in each bin. SNPs with negative scores tend to be heterozygous. (C) Proportion of SNPs found in the other animal's SNP list. SNPs with negative scores are less frequently present in both animals.

In humans and other animals, numerous phenotypes, both Mendelian and quantitative, have been linked to nonsynonymous SNPs [14]. One approach that is used to highlight potentially functionally important nonsynonymous SNPs involves comparing a protein sequence to its orthologues [15,16]. To better characterize the large number of nonsynonymous SNPs identified in this work, we measured the severity of the corresponding amino acid changes by examining orthologous protein sequences in Ensembl [17]. The results were quantified for each nonsynonymous SNP as an "alignment score change" (ASC) value. In short, a negative value arises when the non-reference allele changes the protein so that it no longer resembles its orthologues, and a positive value arises when the non-reference allele changes the protein to make it more similar to its orthologues. The majority of the nonsynonymous SNPs we identified involve minor changes from an evolutionary standpoint (ASC values near zero) (Figure 1A and 1B). There is a trend exhibited by the nonsynonymous SNPs in which those with lower ASC values are less frequently detected as homozygous SNPs (Figure 1C and 1D) and also less frequently shared between the two animals (Figure 1E). This trend can be explained by the non-conservative alleles being less prevalent in the population on average than those that yield a protein that resembles its orthologues.

### CNV identification and validation

**Figure 2:**
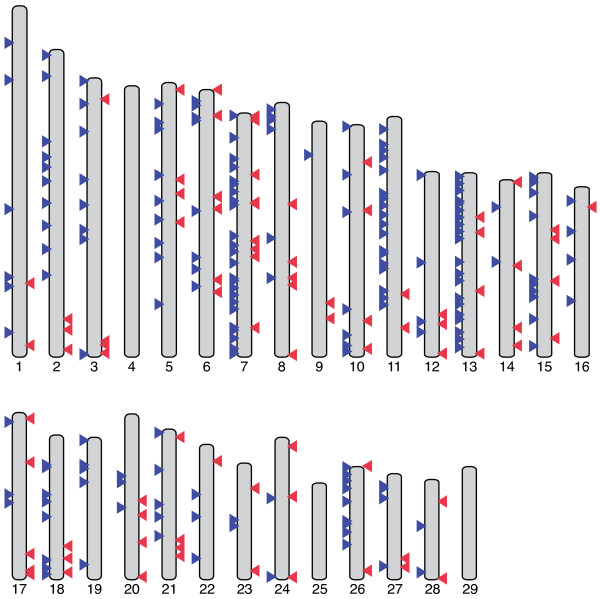
**Genomic distribution of CNVs**. Arrowheads located on the left side of the chromosome ideograms represent CNVs with higher copy number in the Holstein genome (Holstein CNV gains) while arrowheads on the right side in represent CNVs with higher copy number in the Black Angus genome (Black Angus CNV gains). Note that multiple CNVs may appear as a single arrowhead due to their proximity in the genome.

Putative CNVs were detected by identifying regions of the Btau4.0 reference sequence with significantly different coverage between the Holstein and Black Angus mapped read sets [18]. In total, 790 CNVs were identified on the 29 autosomes analysed, involving approximately 3.3 Mbp of the reference assembly used for mapping (~0.13%; Table 6). The average and median CNV sizes are 4,163 bp and 3,171 bp, respectively, and the CNVs range in size from 1,841 bp to 28,029 bp. The CNVs are not evenly distributed along the reference autosomes, with some chromosomes lacking CNVs and others having numerous such regions (Figure 2). The percentage of chromosomal length containing CNVs was less than 1% in all cases and ranged from 0.028% to 0.851% (Table 6). All the CNVs found in this work are described in Additional file 3.

**Table 6:** Summary of CNVs

BTA	Chromosome length	% length in CNV	Total CNV length	No. CNV	Mean length	Median length	Max length	Min length
1	161,106,243	0.028	44,913	11	4,083	4,148	7,151	2,861

2	140,800,416	0.102	143,068	32	4,471	4,865	10,257	2,629

3	127,923,604	0.135	173,057	27	6,410	5,435	28,029	2,861

4	124,454,208	0.000	0	0	0	0	0	0

5	125,847,759	0.246	309,452	51	6,068	6,435	19,448	2,860

6	122,561,022	0.292	358,280	56	6,398	5,610	20,911	2,549

7	112,078,216	0.243	272,175	95	2,865	3,421	9,881	1,901

8	116,942,821	0.163	190,373	37	5,145	5,131	13,111	2,849

9	108,145,351	0.057	61,772	14	4,412	3,949	7,051	2,821

10	106,383,598	0.090	96,123	25	3,845	3,727	8,481	2,569

11	110,171,769	0.075	82,123	31	2,649	2,813	4,499	2,249

12	85,358,539	0.255	217,445	49	4,438	5,005	9,731	2,781

13	84,419,198	0.180	152,086	64	2,376	2,758	3,939	1,969

14	81,345,643	0.113	91,924	26	3,536	3,752	5,628	2,680

15	84,633,453	0.125	106,076	30	3,536	3,611	10,209	2,489

16	77,906,053	0.039	30,688	6	5,115	3,562	14,248	2,740

17	76,506,943	0.048	36,720	11	3,338	3,510	6,480	2,700

18	66,141,439	0.851	563,154	120	4,693	6,100	18,405	2,141

19	65,312,493	0.035	22,660	6	3,777	2,987	6,723	2,489

20	75,796,353	0.036	27,332	8	3,416	3,589	4,141	2,761

21	69,173,390	0.064	44,293	13	3,407	3,568	5,413	2,461

22	61,848,140	0.031	19,408	4	4,852	4,033	8,821	2,521

23	53,376,148	0.095	50,750	12	4,229	3,500	12,000	2,500

24	65,020,233	0.053	34,298	10	3,430	3,307	5,389	2,449

25	44,060,403	0.000	0	0	0	0	0	0

26	51,750,746	0.134	69,401	33	2,103	2,301	2,761	1,841

27	48,749,334	0.144	70,250	14	5,018	4,126	20,071	2,231

28	46,084,206	0.045	20,587	5	4,117	3,943	5,913	2,409

29	51,998,940	0.000	0	0	0	0	0	0

TOTAL	2,545,896,661	0.129	3,288,408	790	4,163	3,171	28,029	1,841

The distribution and size characteristics of CNVs detected through comparison of the Holstein and Black Angus read sets mapped to the Btau4.0 reference assembly.

**Figure 3:**
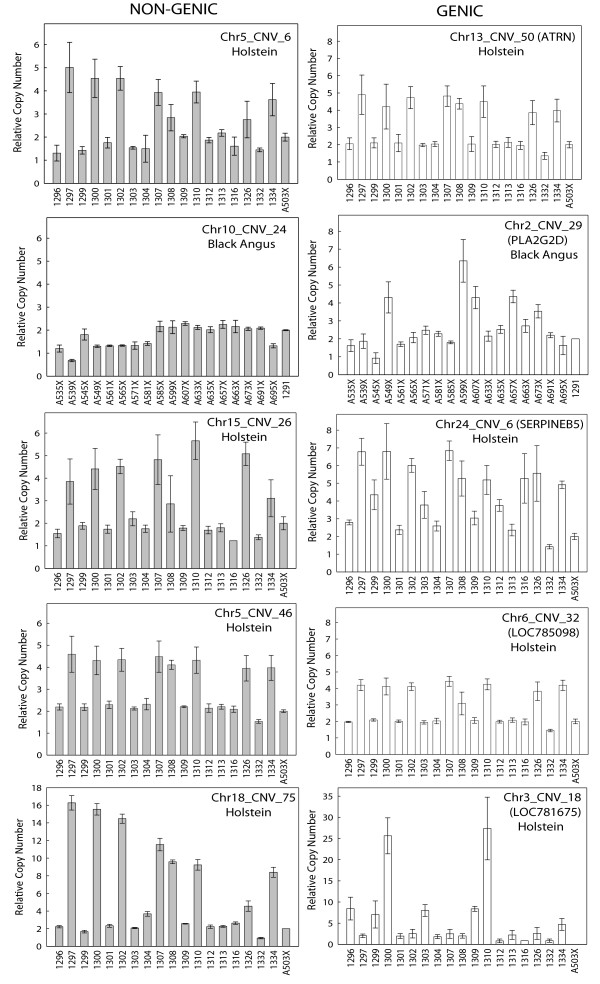
**Validation of CNVs using qPCR**. Validation results for non-genic CNVs (panels on the left) and genic CNVs (panels on the right) are shown. Each panel is labelled with the CNV tested, and the breed assayed. The name of the overlapping gene is given in parentheses for genic CNVs. Bars represent distinct animals and are labelled with animal identifiers. The right-most bar in each panel depicts the relative copy number in a calibrator animal from the alternate breed. The calibrator is assumed to contain two copies of the DNA segment. Each bar was calculated from four technical replicates. The error bars show the minimum and maximum value encountered among the replicates.

Five genic CNVs and five non-genic CNVs (Table 7) were randomly selected for evaluation by quantitative real-time PCR (qPCR). The CNVs identified as gains in the Holstein animal (n = 8) were quantified in 18 Holstein animals and those identified as gains in the Black Angus animal (n = 2) were quantified in 18 Black Angus animals. All ten of the tested regions exhibited copy number differences (Figure 3).

**Table 7:** CNVs selected for validation by qPCR

CNV ID	Entrez Gene	Log_2 _ratio	P-value
Chr2_CNV_29	PLA2G2D1	-1.799	0

Chr3_CNV_18	LOC781675	0.813	3.06E-99

Chr5_CNV_6	-	0.871	3.44E-112

Chr5_CNV_46	-	2.777	0

Chr6_CNV_32	LOC785098	0.811	3.47E-141

Chr10_CNV_24	-	-2.954	0

Chr13_CNV_50	ATRN	0.858	4.00E-171

Chr15_CNV_26	-	0.806	3.79E-109

Chr18_CNV_75	-	0.951	1.64E-138

Chr24_CNV_6	SERPINB5	0.991	8.42E-164

Five genic and five non-genic CNVs were selected for validation by qPCR. Entrez Gene names are given for genic CNVs. The log_2 _ratios and p-values obtained from the CNV-seq software are shown. Positive log_2 _ratios indicate higher read depth in the Holstein animal.

### CNV annotation and Gene Ontology analysis

To identify potential functional roles associated with the putative CNVs, genes completely or partially overlapping with these CNVs were retrieved from Ensembl [17]. A total of 164 genes were identified, involving 250 of the CNVs. Gene Ontology (GO) enrichment analysis [19] indicates that genes related to "response to stimulus" (GO:0050896; P < 0.01), "immune system process" (GO:0002376; P < 0.01), and "growth" (GO:0040007; P < 0.01) are over-represented in the set of CNVs identified in this work (Table 8). The term "locomotion" (GO:0040011; P < 0.01) is enriched among the CNVs with higher copy number in Black Angus, while among the Holstein CNV gains the terms "reproduction" (GO:0000003; P < 0.01), "reproductive process" (GO:0022414; P < 0.01), "membrane-enclosed lumen" (GO:0031974; P < 0.01), and "enzyme regulator activity" (GO:0030234; P < 0.01) are enriched (Table 8).

**Table 8:** Gene Ontology terms enriched among the CNVs

Ontology	GO ID	Description	Animal	P-BA	P-HOL
BP	GO:0032502	Developmental process	Both	4.3E-21	3.5E-40

BP	GO:0032501	Multicellular organismal process	Both	6E-34	3.4E-67

BP	GO:0050789	Regulation of biological process	Both	0.00042	0.00015

BP	GO:0002376	Immune system process	Both	2.4E-19	1.4E-17

BP	GO:0016043	Cellular component organization	Both	1.3E-07	9.7E -12

BP	GO:0065007	Biological regulation	Both	5.5E-05	9.2E-05

BP	GO:0048518	Positive regulation of biological process	Both	1.3E-35	2.6E-29

BP	GO:0048519	Negative regulation of biological process	Both	5.7E-16	1E-30

BP	GO:0022610	Biological adhesion	Both	8.5E-06	0.028

BP	GO:0016265	Death	Both	1.5E-13	2.3E-07

BP	GO:0009987	Cellular process	Both	0.011	0.077

BP	GO:0008152	Metabolic process	Both	0.0051	0.015

BP	GO:0051234	Establishment of localization	Both	0.011	0.00069

BP	GO:0051179	Localization	Both	0.002	3.5E-07

BP	GO:0040007	Growth	Both	9.7E-07	3.8E-16

BP	GO:0050896	Response to stimulus	Both	3.2E-30	9.1E-41

BP	GO:0044085	Cellular component biogenesis	Both	0.00072	0.0044

BP	GO:0040011	Locomotion	BA	5.4E-12	-

BP	GO:0000003	Reproduction	HOL	-	1.6E-25

BP	GO:0022414	Reproductive process	HOL	-	3.8E-18

CC	GO:0032991	Macromolecular complex	Both	0.007	1.6E-05

CC	GO:0005623	Cell	Both	0.0016	0.00025

CC	GO:0044464	Cell part	Both	0.0016	0.00025

CC	GO:0044421	Extracellular region part	Both	1.2E-14	5.8E-13

CC	GO:0005576	Extracellular region	Both	2E-08	7.8E-07

CC	GO:0043226	Organelle	Both	0.0054	4.3E-07

CC	GO:0044422	Organelle part	Both	0.00024	6.8E-10

CC	GO:0031974	Membrane-enclosed lumen	HOL	-	4.5E-06

MF	GO:0060089	Molecular transducer activity	Both	0.013	0.0031

MF	GO:0005215	Transporter activity	Both	0.07	0.43

MF	GO:0003824	Catalytic activity	Both	0.071	0.095

MF	GO:0005488	Binding	Both	0.0015	0.0032

MF	GO:0030528	Transcription regulator activity	HOL	-	0.16

MF	GO:0030234	Enzyme regulator activity	HOL	-	3.6E-09

GO IDs from the three GO ontologies (BP = Biological Process; CC = Cellular Component; MF = Molecular Function) enriched among the CNV gains in Black Angus (BA), Holstein (HOL), or both animals (Both). P-values are provided, when applicable, for the subset of CNVs detected as gains in the Black Angus animal (P-BA) and the subset detected as gains in the Holstein animal (P-HOL).

## Discussion

### SNP list characteristics

The proportions of novel SNPs identified in this work (81% in both Holstein and Black Angus) are very close to the proportion of novel SNPs identified through the sequencing of a Fleckvieh bull genome (82%) [8]. These values suggest that a large number of DNA variants remain to be identified in cattle. The false negative rate of 10% and 21% for homozygous and heterozygous SNPs in the Holstein bull indicates that our work does not provide a comprehensive list of the SNPs in the animals we sequenced, and that further sequencing or modified analysis procedures may be helpful for gaining a more complete picture of the genomes of these animals. The low false-positive rates for both lists indicate that the vast majority of the SNPs we report are true SNPs. The SNPs from both animals are available from dbSNP [13] ([dbSNP:ss411633515] to [dbSNP:ss418635388]).

### SNPs of potential functional significance

SNP annotation aims to provide some indication as to which SNPs may be functionally relevant. Among the nonsynonymous SNPs we identified, those that cause a dramatic protein sequence change from the standpoint of an alignment-scoring matrix (large alignment score change) may be of particular interest. The SNPs that create stop codons can also be imagined to have important effects. This class is enriched for heterozygous SNPs (71% compared to 54% for all SNPs in the Holstein bull and 73% compared to 51% for all SNPs in Black Angus). The annotation tool we used for this work (NGS-SNP) provides the names of protein features that overlap with SNP-altered residues (phosphorylation sites for example), as well as descriptions of protein function, gene names and identifiers, GO information, and known phenotypes in cattle or in humans linked to the SNP-affected gene or its human orthologue. This information, particularly in conjunction with QTL mapping or genome-wide association results, should be useful for future work aimed at better understanding the genetic mechanisms underlying phenotypic differences in cattle.

### Comparison of CNVs to those identified in previous work

Previous studies examining CNVs in cattle have employed array comparative genomic hybridization (aCGH) [9,20] or SNP arrays [21]. Next-generation sequencing has been used previously for CNV detection in humans [22-25]. The sequencing approach can overcome the sensitivity limits of aCGH and SNP arrays, and can more precisely identify CNV boundaries [22].

A substantial number of the CNVs from this work (42%) are concordant with the CNVs previously identified in cattle using aCGH [9]. This concordance with the aCGH findings, in conjunction with the PCR validation results, lends further support to the CNVs described in this study. Differences observed between the CNVs described here and those detected using aCGH can be attributed to the particular breeds investigated and to differences between the technologies used. The CNVs we detected by read-depth analysis are on average much smaller than those identified by aCGH (4,163 bp vs. 203,648 bp) [9]. In human studies, the use of sequencing also led to the identification of much shorter CNVs compared to aCGH [22]. The approach we used to detect CNVs can artificially break a single CNV into multiple CNVs. For example, if read depth happens to drop in one or both of the animals in the middle of a CNV then two CNVs may be reported because the middle region does not meet the criteria for reporting.

### Gene Ontology analysis of CNVs

Gene Ontology enrichment analysis indicates that genes related to "response to stimulus," "immune system process," and "growth" are over-represented in the set of CNVs identified in this work (Table 8). "Response to stimulus" is defined as a change in state or activity of a cell or an organism (in terms of movement, secretion, enzyme production, gene expression, etc.) as a result of a stimulus [19]. "Immune system response" is defined as any process involved in the development or functioning of the immune system: i.e., an organismal system for calibrated responses to potential internal or invasive threats [19]. Genes related to immunity and sensory response have been previously identified as being overrepresented in CNVs in cattle [9] and in humans [26]. It has been suggested that the increased dosage of genes related to infection response and sensing the environment offer a survivability benefit [9,27]. "Growth" is defined as the increase in size or mass of an entire organism or a part of an organism [19]. Perhaps enrichment of this term reflects the selection applied to these breeds, but as noted below none of these CNVs have been specifically associated with traits in cattle to our knowledge.

Each CNV is detected in this work as a gain of sequence dosage in one animal relative to the other. Some GO terms are enriched among the CNV gains in one animal but not among the CNV gains in the other. The GO term "locomotion" is enriched among the CNVs with higher copy number in Black Angus, while among the CNV gains in Holstein the terms "reproduction", "reproductive process", "membrane-enclosed lumen", and "enzyme regulator activity" are enriched. The term "locomotion" is defined as self-propelled movement of a cell or organism from one location to another and includes genes such as myotubularin related protein 9. Enrichment of this term could be imagined to reflect selection for lean muscle mass in beef cattle, while the enrichment of reproduction-related genes (which includes genes related to lactation) in the Holstein animal is consistent with the selection applied to the Holstein breed. At this point however there is no evidence linking the CNVs we detected to increased gene activity or to phenotypic differences.

### A gene of interest overlapping with CNVs

**Figure 4:**
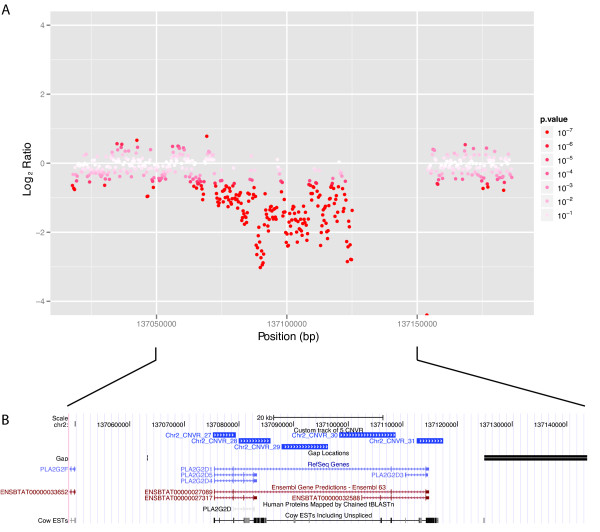
**CNVs overlapping with *PLA2G2D *gene region**. (A) Log_2 _ratio plot of the *PLA2G2D *gene region. Each point shows the log_2 _ratio of the number of Holstein reads mapped to the number of Black Angus reads mapped. Points are coloured based on the log_10 _p-value calculated by the CNV-seq software. (B) The *PLA2G2D *gene region as visualized using the UCSC Genome Browser. The precise boundaries of the five CNVs reported by CNV-seq that reside in this region are shown and labelled. The third CNV from the left (Chr2_CNV_29) was tested and validated by qPCR.

Several CNVs were found to overlap with genes that potentially influence beef or dairy traits of interest, such as milk production, health, or meat quality. For example, CNVs overlapping with a *PLA2G2D *gene were identified (Figure 4). *PLA2G2D *genes are thought to play roles in lipid metabolism, fat deposition, gonadotropin-releasing hormone signalling, and MAPK signalling [28]. The region shown in Figure 4 also underlies QTL for body weight and carcass weight in beef cattle [29]. One of the five CNVs located in the *PLA2G2D *region was quantified using qPCR (we suspect that the five CNVs represent a single CNV that was split due to the limitations of our detection approach). Among the Black Angus animals tested by qPCR, copy number differences were observed relative to the Holstein calibrator (Figure 3). Future work will be needed to establish whether these CNV differences are associated with phenotypic variation.

### Abundance of CNV gains in Holstein

Strong selection has led to impressive performance gains in the Holstein breed, particularly in the past 50 years. It is not possible to assess from our data whether the apparent abundance of CNV gains in the single Holstein animal we examined is related to selection. In other species, natural selection is thought to have favoured the expansion of CNVs that influence certain traits, such as immunity in the case of mice and humans [27]. Further research examining more individuals may allow us to discern whether artificial selection has had a role in shaping CNVs in cattle.

## Conclusions

Whole genome resequencing of a Black Angus bull and a Holstein bull identified 3.2 and 3.7 million SNPs respectively, through comparisons with the Hereford reference sequence. Numerous CNVs were also found through an analysis of read depth differences. Downstream validation suggests a low false-positive rate for SNP and CNV detection, but that many SNPs were likely missed by sequencing. More work is needed to investigate the source and significance of the higher proportion of CNV gains in the Holstein animal. The deeply annotated SNPs and CNVs identified in this resequencing study can serve as useful genetic tools, and as candidates in searches for phenotype-altering DNA differences.

## Methods

### DNA sequencing

Genomic DNA from a Black Angus bull and a Holstein bull was sequenced using the Applied Biosystems SOLiD 3 sequencer (Life Technologies Corporation, CA, USA), using a combination of fragment and mate-paired libraries. The libraries were prepared using the reagents and protocols provided by Applied Biosystems.

Fragment libraries were generated by shearing 6 μg of genomic DNA into small fragments with a mean size of 110 bp using the Covaris S2 system (Covaris, MA, USA) followed by end-repairing the DNA and ligating the P1 and P2 adaptors. The ligated, purified DNA was analyzed on an E-Gel 2% Size-Select gel (Invitrogen, ON, Canada) and 150-200 bp ligation products were collected, nick translated and amplified using library PCR primers 1 and 2. The PCR amplified samples were purified using the PureLink PCR purification kit (Invitrogen, ON, Canada).

For mate-paired library preparation, 40-60 μg of DNA was sheared into 1.5 kb fragments for 2 × 50 bp libraries and 2.5 kb fragments for 2 × 25 bp libraries using a HydroShear (DigiLab Genomic Solutions Inc, MA, USA). The fragmented DNA was end repaired using the End-It™ kit (Epicentre Biotechnologies, MA, USA), and ligated to LMP CAP adapters for 2 × 50 bp libraries and EcoP151 for 2 × 25 bp libraries. The DNA was then size selected by electrophoresis on a 1% agarose gel, recovered from the gel using the PureLink Quick Gel Extraction Kit and the QIAquick Gel Extraction Kit (Qiagen, ON, Canada) and circularized by ligation to a biotinylated internal adapter. The circularized DNA was isolated and digested before binding the library molecules to streptavidin beads. Before amplification of the library using PCR primers 1 and 2, the double-stranded P1 and P2 sequencing adapters were ligated to the end-repaired DNA. The amplified libraries were purified using the PureLink PCR Micro Kit (Invitrogen, ON, Canada).

Average molecule sizes of all the libraries were confirmed by analysis with a Bioanalyzer and a DNA 1000 chip (Agilent, ON, Canada). The final concentrations of all the libraries were measured using the StepOnePlus Quantitation Kit (Life Technologies Corporation, CA, USA) so that appropriate template volumes could be added to emulsion PCR reactions, which were performed using the SOLiD ePCR Kit (Life Technologies Corporation, CA, USA). A portion of the beads was subjected to sequencing to assess the quality and to determine the volume of beads to be used for sequencing.

Massively parallel DNA sequencing was performed using an Applied Biosystems SOLiD System (V3 chemistry) [24]. The fragment libraries were sequenced to 50 bases. For the mate-paired libraries, both forward and reverse tags were sequenced to 25 bases for the 2 × 25 bp libraries and 50 bases for 2 × 50 bp libraries.

### SNP identification

Sequence reads were mapped to the Btau4.0 Bovine genome assembly using the Bioscope 1.0 software suite (Life Technologies Corporation, CA, USA). A list of putative SNPs was generated for each animal from the mapped reads, using the diBayes SNP Detection module (with the "med-coverage" stringency setting) included with Bioscope. The lists were subjected to additional filtering to remove SNPs with particularly high read depth (higher than 95% of the other SNPs from the same animal), and to remove SNPs that could not be unambiguously placed on the improved bovine genome assembly UMD3.1 [30] using the megablast algorithm [31] in BLAST+ [32]. For the BLAST analysis, 100 bp of flanking sequence was used along with the default program settings, except that an E-value threshold of 1E-35 was specified using the "-evalue" option, and query sequence filtering was disabled using the "-dust no" setting.

### Evaluation of sequencing-derived SNPs by genotyping using an existing array

To estimate the completeness of the SNP lists obtained by sequencing, the Holstein bull was genotyped using the BovineHD BeadChip (Illumina Inc., CA, USA). Before comparing the genotyping results to the sequencing SNPs, BLAST was used to position each of the BovineHD SNPs on the Btau4.0 assembly. A total of 711,765 BovineHD SNPs could be unambiguously placed on the reference chromosomes used for read mapping (the 29 autosomes and chromosome X). These SNPs were used in subsequent comparisons with the sequencing SNPs. SNPs successfully genotyped using the BovineHD array and that were not homozygous for the reference alleles were compared to the sequence-derived SNPs. The false negative rate was calculated for homozygous sequence-derived SNPs as the percentage of non-reference-allele homozygous SNPs identified by genotyping that were not concordantly called as SNPs by sequencing (i.e., were missed altogether or were assigned alleles inconsistent with the genotyping). The false negative rate for heterozygous SNPs was calculated in a similar fashion, as the percentage of array-based heterozygous calls that were not concordantly called as SNPs by sequencing. SNPs identified by both sequencing and genotyping but which did not agree in terms of alleles present (discordant SNPs) were further classified based on the nature of the discrepancy.

### Estimation of the false-positive rate of SNP discovery

An Infinium iSelect HD Custom Beadchip (Illumina, San Diego CA) was used to genotype 427 SNPs and 422 SNPs selected from the Holstein and Black Angus SNP sets, respectively. DNA for genotyping was obtained from 1083 steers at the University of Guelph. GeneSeek (Lincoln, NE) performed the genotyping and SNP calls were made using the GenomeStudio software package (Illumina, San Diego CA). The rate of false-positive discovery was calculated as the number of monomorphic SNPs returned, divided by the total number of SNPs.

### SNP annotation

NGS-SNP [12] was used to assign a functional class to each SNP and to provide several fields of information describing the affected transcript and protein, if applicable (Additional file 4). The source databases used during the annotation include Ensembl release 57 [17], dbSNP build 130 (consisting of 2,210,483 bovine refSNP SNPs) [13], Entrez Gene [13], and UniProt release 2010_12 [33]. For non-synonymous SNPs, an "alignment score change" (value *a*) was calculated by comparing the reference amino acid and the non-reference amino acid to each orthologue. Briefly, the amino acid encoded by the variant (i.e., non-reference) allele *v *is compared to each available orthologous amino acid *o *using a log-odds scoring matrix (BLOSUM62). Similarly, the amino acid encoded by the reference allele *r *is compared to the orthologues. The final score is the average score for the variant amino acid minus the average score for the reference amino acid (1). A positive value indicates that the variant amino acid is more similar to the orthologues than the reference amino acid, whereas a negative value indicates that the reference amino acid is more similar to the orthologues.

(1) $$a=\frac{\underset{o}{\sum }s\left(v,o\right)}{n}-\frac{\underset{o}{\sum }s\left(r,o\right)}{n}$$

### CNV identification

Putative CNVs on the 29 bovine autosomes were identified using the CNV-seq program, which examines the mapped reads from two individuals and reports regions that exhibit statistically significant read depth differences [18]. Briefly, the Holstein and Black Angus reads were mapped to the Btau4.0 reference assembly [6] using the Bioscope 1.0 software suite (Life Technologies Corporation, CA, USA). The output of Bioscope was converted into the "best-hit" format required by CNV-seq using the best-hit.SOLiD.pl Perl script. The cnv-seq.pl script was then run using the default threshold values (p-value = 0.001 and log_2 _threshold = 0.6) and a window size setting of 2, to generate a list of CNVs from the best-hit files. A "minimum-windows-required" setting of 10 was used to specify that ten consecutive sliding windows exhibiting a significant read depth difference were required for a region to be annotated as a CNV.

### Quantitative PCR validation of CNVs

Quantitative real-time PCR was performed for CNV validation using the StepOne Plus Real-Time PCR System and the SDS 2.2 software package (Life Technologies Corporation, CA, USA). Primers and probes (Additional file 5) were designed for five genic and five non-genic CNVs using the GeneAssist Copy Number Assay Workflow Builder software (Life Technologies Corporation, CA, USA). All primers were validated by standard curve analysis using a serial dilution of genomic DNA from a common reference animal, with amplification efficiencies above 90% and no-template control reactions. All reactions (20 μL) were run in quadruplicate with 1X TaqMan Genotyping PCR Master Mix (Life Technologies Corporation, CA, USA), 1 μL of 20 × working stock of TaqMan Copy Number Assays (Life Technologies Corporation, CA, USA) for target genes, 100 nM of each primer, 250 nM probe for the reference genes and 40 ng of genomic DNA. Thermal-cycling conditions were as follows: 95°C for 10 minutes followed by 40 cycles at 95°C for 15 seconds and 60°C for 60 seconds. An average ΔCt for each sample (from four replicates) was calculated after normalizing to BTF3 (chr20:8480505-8487056 on Btau4.0) [9]. The copy number of each target gene was calculated using the CopyCaller software (Life Technologies Corporation, CA, USA) based on the assumption that there were two copies of the DNA segment in the calibrator animals. The relative quantification analysis of each target assayed in Holstein or Black Angus animals was performed using a Black Angus or Holstein animal, respectively, as a calibrator. One CNV (Chr3_CNV_18) could not be calibrated due to the absence of the sequence in the Black Angus animals tested. Instead, the most frequently observed copy number among the Holstein animals was assumed to represent two copies.

### CNV annotation and Gene Ontology analysis

A custom Perl script was used to conduct a search of Ensembl (release 67) [17] for each CNV identified using CNV-seq to identify overlapping genes. The canonical transcript record for each overlapping gene was used to obtain an Ensembl protein ID, and the set of IDs was analyzed using the agriGO server's Singular Enrichment Analysis (SEA) tool [34] to identify GO terms enriched among the CNVs. Fisher's exact test was used to assess the significance of term enrichments, as recommended by the authors, and the default multiple comparison correction (Benjamini-Yekutieli method) was applied.

## Authors' contributions

PS drafted the manuscript with assistance from the other authors, wrote software for SNP annotation, and determined the false-negative rate of SNP detection. JC identified CNVs, performed Gene Ontology analyses, and determined the false-positive rate of SNP detection. UB and YM performed library construction and DNA sequencing and UB assisted with validation of SNPs and CNVs. JMS performed genotype data interpretation and performed CNV validation. XL assisted with SNP annotation. SSM conceived the study and assisted with data interpretation. All authors have read and approved the final manuscript.

## Supplementary Material

Additional file 1 **Annotated Holstein SNPs**. Tab-delimited text file of Holstein SNPs identified and annotated in this work. Intergenic and intronic SNPs were removed to reduce the size of the file. Click here for file

Additional file 2 **Annotated Black Angus SNPs**. Tab-delimited text file of Black Angus SNPs identified and annotated in this work. Intergenic and intronic SNPs were removed to reduce the size of the file. Click here for file

Additional file 3 **CNV details**. Tab-delimited text file describing CNVs detected in this work. Click here for file

Additional file 4 **SNP annotation fields**. PDF file containing a table of SNP annotation column descriptions. Click here for file

Additional file 5 **CNV validation primers and probes**. PDF file containing a table of CNV validation primers and probes. Click here for file
