# Supplementary material for: Whole genome resequencing of black Angus and Holstein cattle for SNP and CNV discovery
Source: BMC Genomics. 2011 Nov 15;12:559. doi: 10.1186/1471-2164-12-559 (PMC3229636; doi:10.1186/1471-2164-12-559)
Supplement: Additional file 4 — SNP annotation fields. PDF file containing a table of SNP annotation column descriptions. [file 1471-2164-12-559-S4.PDF]

Additional file 4 - SNP annotation fields

Annotations provided for the SNPs identified in this work. Note that some annotations are only defined for certain SNP types.

Field	Description
Function_Class	Type of SNP (3PRIME_UTR, INTRONIC, etc.)
Chromosome	Chromosome containing the SNP
Chromosome_Position	Position of the SNP on the chromosome
Chromosome_Strand	Strand corresponding to the reported alleles
Chromosome_Reference	Base found in the reference genome
Chromosome_Reads	Base in genome supported by the reads
Gene_Description	Short description of the relevant gene
Ensembl_Gene_ID	Ensembl Gene ID of the relevant gene
Entrez_Gene_Name	Entrez Gene name of the relevant gene
Entrez_Gene_ID	Entrez Gene ID of the relevant gene
Ensembl_Transcript_ID	Ensembl Transcript ID of the affected transcript
Transcript_SNP_Position	Position of the SNP on the transcript
Transcript_SNP_Reference	Base found in the reference transcript
Transcript_SNP_Reads	Base in transcript according to the reads
Transcript_To_Chromosome_Strand	Chromosome strand matching transcript
Ensembl_Protein_ID	Ensembl Protein ID of the affected protein
UniProt_ID	UniProt ID of the relevant protein
Amino_Acid_Position	Position of the affected amino acid
Overlapping_Protein_Domains	Protein domains that overlap with the affected amino acid
Overlapping_Protein_Features	Protein features, obtained from UniProt, that overlap with the affected amino acid
Amino_Acid_Reference	Amino acid encoded by the reference
Amino_Acid_Reads	Amino acid encoded by the reads
Amino_Acids_In_Orthologues	Amino acids from orthologous sequences that align with the reference amino acid
Alignment_Score_Change	Effect of SNP on protein conservation
C_blosum	Conservation score when reference amino acid compared to orthologues using an amino acid scoring matrix
C_ident	Percentage of aligned amino acids in orthologous sequences that are identical to the reference amino acid
Context_Conservation	Average percent identity of the SNP region
Orthologue_Species	Source species of the orthologues used for previous five columns

Gene_Ontology	Gene Ontology IDs and terms for relevant transcript
Model_Annotations	Additional functional information obtained by transferring SNPs to human sequences
Ref_SNPs	NCBI rs IDs of known SNPs sharing alleles with this SNP
Is_Fully_Known	Whether existing SNP records completely describe this SNP
