# Supplementary material for: Whole genome resequencing of black Angus and Holstein cattle for SNP and CNV discovery
Source: BMC Genomics. 2011 Nov 15;12:559. doi: 10.1186/1471-2164-12-559 (PMC3229636; doi:10.1186/1471-2164-12-559)
Supplement: Additional file 5 — CNV validation primers and probes. PDF file containing a table of CNV validation primers and probes. [file 1471-2164-12-559-S5.PDF]

Additional file 5 - Primers and probes used for CNV validation by qPCR

The CNV ID can be used to obtain more information about the CNV from Additional file 3. The last row provides the primer and probe sequences used for the BTF3 gene, which was used for normalization (see Methods).

CNV ID	Forward primer	Reverse primer	Probe
Chr2_CNV_29	GCCAAAGCCATGAGACAAATATCTT	TGCATTAATTTGCACAGGCTGATTT	TCGACTCTTCAGGCAGATCAG
Chr3_CNV_18	CACCCGCGTCTCTGGAAAA	GGAACTCTCGATTACTGGACAGAAG	CCATCCCTCCACAATGTTCA
Chr5_CNV_6	TGACCAAGAACACAAAACATAATTCAGATACT	TGGGAATGGCTCAATAGCATGAATA	ATTTTCCCCCCATTGGTTTTCAA
Chr5_CNV_46	GGCTAGGTGGTTGAGAAAGTCTATT	ACGATGAGCCAGTTGTCAACAAA	ACAAAGCCTTGACCCTAAAGTAT
Chr6_CNV_32	ACCCTGCGATGATTTGATGAGAAG	AAATGTTCTGTGTGAATACGCTTTTGT	ACTGAGCTCTTCACTGGTTTTC
Chr10_CNV_24	CCAGAAAGCCTTGGGAGGTT	CTCTGTCCCTGACTGCTATGC	CTGGCTTGAGGCATTTTCCACC
Chr13_CNV_50	AAGGAAAAGATCCACACAAATTCAAAACA	CCCAGGAAGATGACCGCTATT	ACACAGCCATCAATTTTG
Chr15_CNV_26	GCACTGTAGCACTGAACTCTATTGA	GCATAGTAATAGGAACAGACTCCAAGG	TTGAGAATCCAAATGAACAAATAAATTC
Chr18_CNV_75	AACAGACTTAGTACATAGACAGTGAGACA	ACTCCCTTCTCATGGCATCCA	CTTCCCAGATACCAGGGTCCTAC
Chr24_CNV_6	CACGCACGATAAAACATCACATTCA	CCAGCAGCTCTGCGCTAA	CAGGAGTCCTCTCTGAAAATA
-	GGTAAGTCAGTCACCCTGAGCAA	AAGCTCTGCCCAAACAATGTG	ACCGCTACTAGCAAAC
